# Cost-effective downstream processing of recombinantly produced pexiganan peptide and its antimicrobial activity

**DOI:** 10.1186/s13568-018-0541-3

**Published:** 2018-01-24

**Authors:** Baode Sun, David Wibowo, Anton P. J. Middelberg, Chun-Xia Zhao

**Affiliations:** 1 Australian Institute for Bioengineering and Nanotechnology, The University of Queensland, St Lucia, QLD 4072 Australia; 2 Faculty of Engineering, Computer, and Mathematical Sciences, The University of Adelaide, Adelaide, SA 5005 Australia

**Keywords:** Selective precipitation, Recombinant *E. coli*, Antimicrobial peptides, Carrier proteins, Protein purification, Minimum bactericidal concentration

## Abstract

Antimicrobial peptides (AMPs) have significant potential as alternatives to classical antibiotics. However, AMPs are currently prepared using processes which are often laborious, expensive and of low-yield, thus hindering their research and application. Large-scale methods for production of AMPs using a cost-effective approach is urgently required. In this study, we report a scalable, chromatography-free downstream processing method for producing an antimicrobial peptide, pexiganan, using recombinant *Escherichia coli* (*E. coli*). The four helix bundle structure of the unique carrier protein DAMP4 was used to facilitate a simple and cheap purification process based on a selective thermochemical precipitation. Highly pure fusion protein DAMP4_var_-pexiganan was obtained at high yield (around 24 mg per 800 mL cell culture with a final cultivation OD_600_ ~ 2). The purification yield of DAMP4_var_-pexiganan protein is increased twofold with a 72.9% of the protein recovery in this study as compared to the previous purification processes (Dwyer in Chem Eng Sci 105:12–21, 2014). The antimicrobial peptide pexiganan was released and activated from the fusion protein by a simple acid-cleavage. Isoelectric precipitation was then applied to separate the pexiganan peptide from the DAMP4_var_ protein carrier. The final yield of pure bio-produced pexiganan was 1.6 mg from 800 mL of bacterial cell culture (final cultivation OD_600_ ~ 2). The minimum bactericidal concentration (MBC) test demonstrated that the bio-produced pexiganan has the same antimicrobial activity as chemically synthesized counterpart. This novel downstream process provides a new strategy for simple and probable economic production of antimicrobial peptides.

**Electronic supplementary material:**

The online version of this article (10.1186/s13568-018-0541-3) contains supplementary material, which is available to authorized users.

## Introduction

Over the past few decades, the vast number of studies on antibiotics has greatly stimulated the discovery and development of modern medicines. However, the utility of antibiotic drugs has been increasingly viewed as limited, and potentially harmful long-term due to their tendency to cause bacterial resistance (Andersson et al. 2000). In recent years, antimicrobial peptides (AMPs) has received significant attention as an ideal alternative to classical antibiotics because of their unique antimicrobial properties (Fox 2013). AMPs are a class of short peptides (< 100 amino acids) secreted by living organisms in their epithelial layers, phagocytic cells, or bodily fluids, and provide innate immunity against parasitization by potentially harmful microbes (Zasloff 2002). The antimicrobial properties of AMPs as well as their mechanisms of action have been documented against a broad spectrum of pathogenic microorganisms (Brogden 2005). AMPs can effectively disrupt bacterial cell membranes causing a quick leakage of the intracellular materials (Matsuzaki et al. 1995a). The rapid antimicrobial activity and quick killing effect of AMPs make it difficult for microorganisms to develop resistance against AMPs (Guaní-Guerra et al. 2010). In addition, many AMPs exhibit toxicity against prokaryotic cells but not against eukaryotic cells such as human cells due to different components of their cell membranes (Lee et al. 2015; Matsuzaki et al. 1995b). Despite the promising applications of AMPs, the production of AMPs suffers from limitations in cost and yield, a common problem for those products isolated from natural sources. Therefore, it is necessary to develop a sustainable, scalable and low-cost method to produce AMPs for further research development and for medical application.

Typical methods for producing AMPs are based on chemical and biotechnological approaches. The chemical method is a rapid approach for the production of custom-designed peptides in small quantities (Andersson et al. 2000; Merrifield 1963). However, the process employs toxic chemicals and is not generally economically feasible especially for process scale-up of peptides with a sequence length over 35 amino acids (Latham 1999). Alternatively, peptides can be renewably produced by bio-synthesis methods using microbial cell factories (Li 2011). Simple and process-relevant prokaryotic hosts such as *Escherichia coli* (*E. coli*) can be utilized as a microbial platform to intracellularly express AMPs rather than using expensive and complex eukaryotic hosts like insect and yeast cells (Sørensen and Mortensen 2005). However, the intrinsic toxicity of AMPs induces their proteolysis in host cells and inhibits cell growth, thus limiting expression yield. In addition, many positively-charged AMPs strongly interact with anionic cellular components in the host cells, such as DNA or membrane lipid, leading to difficulty in the purification of AMPs (Li 2009). Protein fusion strategies have been explored to increase the stability and solubility of the AMPs in host cells (Chen et al. 2008; Young et al. 2012). These strategies are carried out by combining an AMP with a carrier protein through a linker that provides a site recognition for cleavage of the AMP from the fusion protein. Downstream processes generally involve recovery and purification of the soluble fusion proteins from the host cells, and subsequent cleavage of the AMPs from the fusion proteins and polishing to high product purity (Ingham and Moore 2007; Li 2011; Parachin et al. 2012). However, most downstream processing methods mainly rely on expensive chromatographic approaches to purify the fusion proteins, which lower the overall protein yield substantially (Azevedo et al. 2009). Also, peptide cleavage uses polluting chemicals (e.g., cyanogen bromide (Zhou et al. 2005)) or expensive enzymes (e.g., enterokinase (Huang et al. 2006)).

Our group designed and developed a novel four-helix bundle protein DAMP4 [MD(PSMKQLADS-LHQLARQ-VSRLEHAD)_4_] (Middelberg and Dimitrijev-Dwyer 2011). In addition to its surface activity, DAMP4 protein can be easily produced in recombinant *E. coli* at high-expression yield and high solubility in a simple buffer, and DAMP4′s four-helix bundle structure in bulk solution makes it stable and soluble under high temperature and high salt concentration (Dwyer et al. 2014). These unique properties allow the high-yield purification of DAMP4 proteins with high purity by using precipitation-based methods (Dwyer et al. 2014; Zhao et al. 2015).

Recently, we used a variant of DAMP4 protein as a carrier to the antimicrobial pexiganan peptide (Zhao et al. 2015). The fusion protein DAMP4_var_-pexiganan [M(EPS MKQLADSLHQLARQVSRLEHA)_4_ DPS GIGKFLKKAKKFGKAFVKILKKHH] has a molecular weight (*MW*) of 14.1 kDa and an isoelectric point (p*I*) of 10.7 (Zhao et al. 2015). Pexiganan (GIGKFLKKAKKFGKAFVKILKK) is a cationic peptide and shows attractive antimicrobial characteristics against both Gram-negative and Gram-positive bacteria (Ge et al. 1999). The four-helix bundle structure of DAMP4_var_ was shown capable of protecting pexiganan peptide from degradation when the fusion protein was expressed in the *E. coli* system, and allowed for introduction of process-relevant features to the fusion protein to simplify purification. The D-P-S linker allowed a simple cleavage method to release pexiganan from the fusion protein due to the deamidation reaction at the D-P site under conditions of low pH and high temperature (Hamada and Swanson 1994). In our previous work, we reported that this fusion protein can be used to produce pexiganan via the same purification method as for DAMP4 with acid-cleavage (Zhao et al. 2015). However, only a small amount of pexiganan at a low purity was obtained using the published approach.

In this work, we developed a new downstream process to produce pexiganan peptide with high purity and high yield. This work builds upon the purification process of another DAMP4-based fusion protein, D4S2 (Wibowo et al. 2017). This novel method consists of (i) purification of fusion proteins based on the selective thermochemical precipitation, (ii) the acid-cleavage of fusion proteins to release the peptides and (iii) separation of targeted peptides via isoelectric precipitation. Our method results in a yield of bio-produced and purified pexiganan of around 1.6 mg from 800 mL bacterial cell culture (final cultivation OD_600_ ~ 2), which accounts for 31% recovery of the theoretical yield of pexiganan. This recovery is twice the recovery obtained from the previous methods producing antimicrobial peptides which have similar physicochemical properties (Jang et al. 2009).

## Materials and methods

### Materials

Synthetic pexiganan peptide (GIGKFLKKAKKFGKAFVKILKK, *MW* 2477.19 Da) was custom synthesized by Genscript Corporation (Piscataway, NJ) with purity > 99%. Poly(ethyleneimine) (PEI) 50% (w/v) in water was purchased from Sigma-Aldrich (#P3143, Castle Hill, Australia). A stock solution of PEI 5% (w/v) at pH 8 was prepared by adding hydrochloric acid (HCl). Water with > 18.2 MΩ cm resistivity was obtained from a Milli-Q system with a 0.22 µm filter (Millipore, North Ryde, Australia). All chemicals were of analytical grade obtained from either Sigma-Aldrich or Merck (Frenchs Forest, Australia) and were used as received unless otherwise stated.

### Expression of DAMP4_var_-pexiganan protein

Recombinant plasmid pET-48b(+) comprising a nucleotide sequence encoding DAMP4_var_-pexiganan protein (GenBank Accession Number: MG029580) (Protein Expression Facility, The University of Queensland) was transformed into chemically competent *E. coli* strain BL21(DE3) (Novagen Merck Bioscience, Darmstadt, Germany). The cells were streaked on a Luria–Bertani (LB) agar plate (15 g/L agar, 10 g/L tryptone, 5 g/L yeast extract, 10 g/L NaCl) and then incubated at 37°C overnight. A single colony selected from the plate was inoculated into 5 mL LB media (10 g/L tryptone, 5 g/L yeast extract, 10 g/L NaCl), followed by incubation at 37 °C, 180 rpm (Ratek, Boronia, Australia) overnight. Overnight culture (800 µL, OD_600_ ~ 2.5) was added into 800 mL of 2 × yeast extract and tryptone (2YT) media (16 g/L tryptone, 10 g/L yeast extract, 5 g/L NaCl) and then incubated at 37 °C, 180 rpm until OD_600_ ~ 0.5. Protein expression was induced by adding isopropyl-*b*-d-thiogalactopyranoside (Astral Scientific, Taren Point, Australia) to a final concentration of 1 mM, and cells were further incubated at 37 °C, 180 rpm for 4 h (final OD_600_ ~ 2). Cells were harvested by centrifugation (4000×*g*, 4 °C, 20 min) and cell pellets were stored at − 20 °C for subsequent use. All bacterial-growth media were supplemented with 50 mg/L of kanamycin sulfate.

### Optimization of DAMP4_var_-pexiganan purification

Cell pellet was resuspended in 40 mL of lysis buffer (25 mM Tris–HCl, pH 8) containing NaCl, and cells were lysed by sonication (Branson Ultrasonics, Danbury, CT) at an energy output of 60 W for 4 burst of 30 s and interspersed in an ice bath for 60 s.

DNA contaminants were removed from crude cell lysates by optimizing concentrations of NaCl and PEI added. To optimize NaCl concentration, NaCl (solid) was added into aliquots of cell lysate to final concentrations varied from 0.2 to 2 M, followed by addition of PEI to a final PEI concentration of 0.5% (w/v) with stirring at 4 °C for 60 min. To optimize PEI concentration, aliquots of PEI solution were added into aliquots of cell lysate containing 1 M NaCl to give final PEI concentrations ranging from 0.05 to 0.5% (w/v) with stirring at 4 °C for 60 min.

After removal of DNA from crude cell lysate, protein solution was collected by centrifugation (38,000×*g*, 4 °C, 20 min). Na_2_SO_4_ (solid) was added into aliquots of the supernatant to final concentrations ranging from 0.2 to 1 M with stirring at 90 °C for 30 min to optimize the precipitation of protein contaminants.

Subsequently, suspension was centrifuged (38,000×*g*, 25 °C, 20 min), and the resulting supernatant was added with Na_2_SO_4_ (solid) to give final Na_2_SO_4_ concentrations ranging from 1.6 to 2.4 M, followed by stirring at 35 °C for 60 min to allow optimization of the selective precipitation of DAMP4_var_-pexiganan protein.

The precipitate of DAMP4_var_-pexiganan protein was recovered by centrifugation (38,000×*g*, 35 °C, 20 min), and then washed with rinsing buffer (25 mM Tris–HCl, 1 M NaCl, pH 8) containing Na_2_SO_4_ at a concentration that retained the protein as a precipitate. Following centrifugation (38,000×*g*, 25 °C, 20 min), the precipitate was resuspended in solubilizing buffer (25 mM Tris–HCl, 1 M NaCl, pH 8), and then dialyzed against 4-(2-hydroxyethyl)-1-piperazineethanesulfonic acid (HEPES) buffer (25 mM, pH 7.5) using a dialysis tubing with SnakeSkin^®^ pleated dialysis membrane (Thermo Fisher Scientific, North Ryde, Australia) with a molecular weight cut-off of 3.5 kDa. The protein solution after buffer exchange was subjected to the peptide-cleavage process.

### Cleavage of pexiganan peptide from the DAMP4_var_ protein carrier

HCl was added to the purified DAMP4_var_-pexiganan protein to a final pH of 4, and then incubated at 60 °C with incubation times varied from 1 to 48 h to optimize the cleavage of pexiganan peptide from DAMP4_var_-pexiganan protein. Subsequently, an aqueous solution of 2 M NaOH was added into the solution (final pH 6.8) and incubated at room temperature for 30 min to induce the precipitation of cleaved DAMP4_var_ protein (theoretical p*I* 6.8). Supernatant containing pexiganan peptide was collected by centrifugation (38,000×*g*, 4 °C, 10 min), and then desalted against water by using an ÄKTA Explorer 10 system equipped with a 5-mL column of Sephadex G-25 resin (GE Healthcare, UK).

### Analytical characterization

To determine DNA concentration, the protein samples (100 µL) were mixed with 1× SYBR^®^ Safe (100 µL) (Life Technologies, Mulgrave, Australia) in a 96-well plate (Greiner Bio-One, Frickenhausen, Germany) for 5 min in a dark room, and then fluorescence intensities were acquired using Infinite^®^ M200 Pro microplate reader (Tecan, Männedorf, Switzerland) at excitation and emission wavelengths of 502 and 530 nm, respectively. A standard curve of DNA (0–50 ng/mL) was constructed using the recombinant plasmid which was extracted and purified from *E. coli* XL1-Blue by using PureLink™ Quick Plasmid Miniprep (Thermo Fisher Scientific, North Ryde, Australia).

Protein samples were qualitatively analyzed by sodium dodecyl sulfate poly(acrylamide) gel electrophoresis (SDS-PAGE) using NuPAGE 4–12% Bis–Tris Precast Gels (Life Technologies, Mulgrave, Australia) mounted in a Bio-Rad XCell 3 system (Bio-Rad, Hercules, CA) with an aqueous buffer solution of 2-(*N*-morpholino)ethanesulfonic acid. Novex™ Sharp Pre-stained Protein Standard (Invitrogen, Carlsbad, CA) was used for the protein ladder.

Concentrations of proteins and peptides were quantitatively determined using reversed-phase high-performance liquid chromatography (RP-HPLC) equipped with a Jupiter C_18_ column (5 µm, 300 Å, 150 mm × 4.6 mm) (Phenomenex, Torrance, CA) and connected to an LC-10AVP series HPLC system (Shimadzu, Kyoto, Japan). Buffer A was 0.1% (v/v) trifluoroacetic acid (TFA) in water, and buffer B was 90% (v/v) acetonitrile and 0.1% (v/v) TFA in water. A linear gradient from 30 to 65% of buffer B was applied at a flow rate of 1 mL/min in 35 min and a detection wavelength was set at 214 nm.

### Antimicrobial activity assay

The antimicrobial activity of the bio-produced pexiganan peptide was determined as compared to that of the controls (water, DAMP4 protein, DAMP4_var_-pexiganan protein, and synthetic pexiganan peptide) by using the minimum bactericidal concentration (MBC) method (Hu et al. 2010). Briefly, a single colony of *E. coli* ATCC^®^ 25922™ (Manassas, VA) selected from a freshly streaked plate was inoculated into 5 mL Mueller-Hinton (MH) Broth (Becton–Dickinson, Sparks, MD) at 37 °C, 180 rpm, and then harvested at the exponential growth phase (OD_600_ ~ 0.5). After rinsing the cells twice by centrifugation (4000×*g*, 4 °C, 20 min), a standard cell suspension was prepared by resuspending the cell pellet in 0.9% NaCl solution to a final concentration of 10^7^ colony-forming units (CFU) per mL (OD_600_ ~ 0.08). Protein/peptide samples (at final concentrations ranging from 1 to 32 µg/mL) as well as water were added into the standard cell suspensions to a final volume of 2 mL. Following incubation at 37 °C, 180 rpm for 2 h, the mixtures (diluted 10,000× in sterilized water) were each spread onto MH agar plates (MH Broth, 1.5% agar) and then incubated at 37 °C for overnight. The percentages of viable cells grown on the agar plates containing the protein/peptide samples were determined by counting the number of the colonies in comparison with the controls.

## Results

In order to obtain DAMP4_var_-pexiganan protein from *E. coli* cells, protein was recovered from cells by sonication, and the released protein was then purified by three main consecutive steps: (1) precipitation of DNA; (2) precipitation of protein contaminants; and (3) isolation of DAMP4_var_-pexiganan protein.

### Removal of DNA

**Fig. 1:**
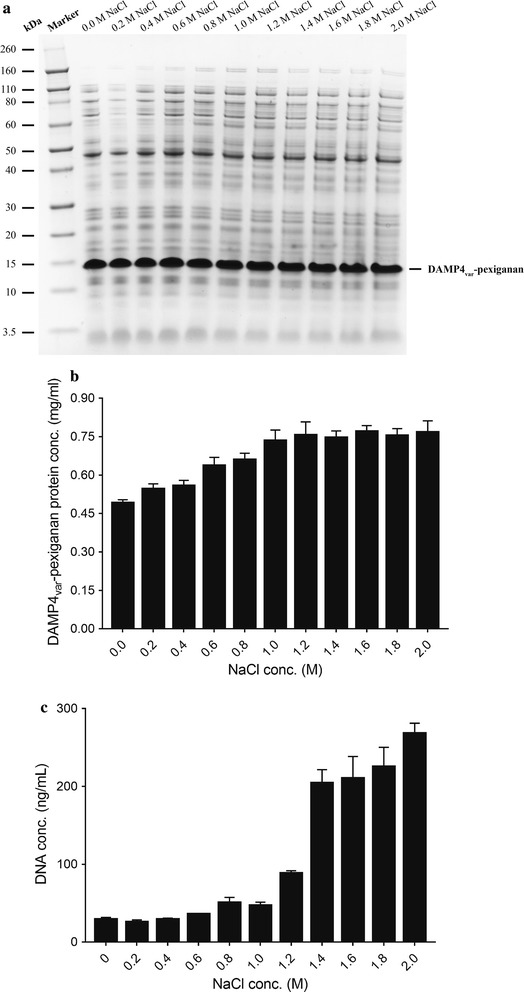
Effect of NaCl concentrations (0–2 M) on the DNA precipitation using poly(ethyleneimine) (PEI) 0.5% (w/v) in Tris–HCl buffer (25 mM, pH 8). **a** SDS–PAGE analysis. Concentrations of (**b**) DAMP4_var_-pexiganan protein and **c** DNA remaining in the solution after PEI addition

In this work, poly(ethyleneimine) (PEI) was used to precipitate DNA from crude cell lysates, and the PEI concentrations were optimized as a function of NaCl concentrations. Figure 1 shows the effects of NaCl concentrations (0–2 M) on precipitating DNA at the PEI concentration of 0.5% (w/v). As qualitatively shown in the SDS-PAGE gel (Fig. 1a), the amount of total soluble protein increased when the NaCl concentrations were increased to 1 M but remained unchanged by further increasing the NaCl concentration to 2 M. Quantitatively, addition of NaCl affected the concentrations of DAMP4_var_-pexiganan protein (Fig. 1b) and DNA (Fig. 1c) in the solutions. The concentration of soluble DAMP4_var_-pexiganan in the presence of 1 M NaCl was 49% higher than that in the absence of NaCl (Fig. 1b). Meanwhile, the concentration of soluble DNA showed gradual increase with increasing NaCl concentration (Fig. 1c). NaCl with a concentration of 1 M was selected for future work as a high removal percentage of DNA and a low protein loss were obtained.

**Fig. 2:**
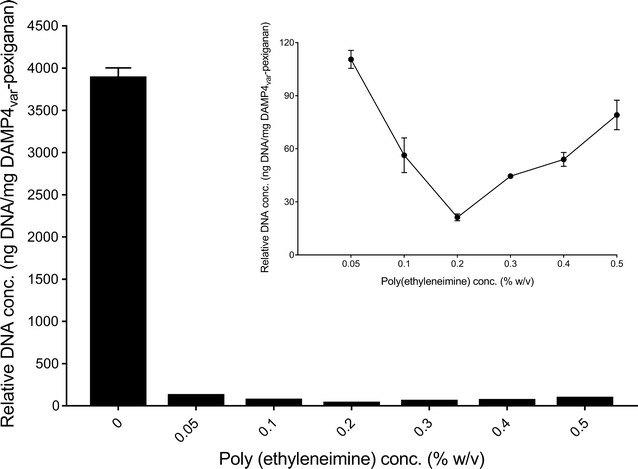
Effect of poly(ethyleneimine) (PEI) concentrations (0.05–0.5% (w/v)) on the DNA precipitation in Tris–HCl buffer (25 mM, pH 8) containing 1 M NaCl

The effect of PEI concentrations on the precipitation of DNA at a NaCl concentration of 1 M was shown in Fig. 2. Comparison of DNA concentrations after treating with PEI to the initial DNA concentration in the absence of PEI revealed that PEI had a significant effect on precipitating DNA. The DNA concentrations decreased when the PEI concentrations were increased from 0 to 0.2%. Approximately 95% of DNA was precipitated at a PEI concentration of 0.05%. At a PEI concentration of 0.2%, the DNA concentration decreased by 99% to 21.3 ng DNA/mg DAMP4_var_-pexiganan protein. However, the DNA concentrations in bulk solution increased when the PEI concentrations were further increased from 0.2 to 0.5%. Therefore, PEI concentration of 0.2% was used for further study.

### Precipitation of protein contaminants

**Fig. 3:**
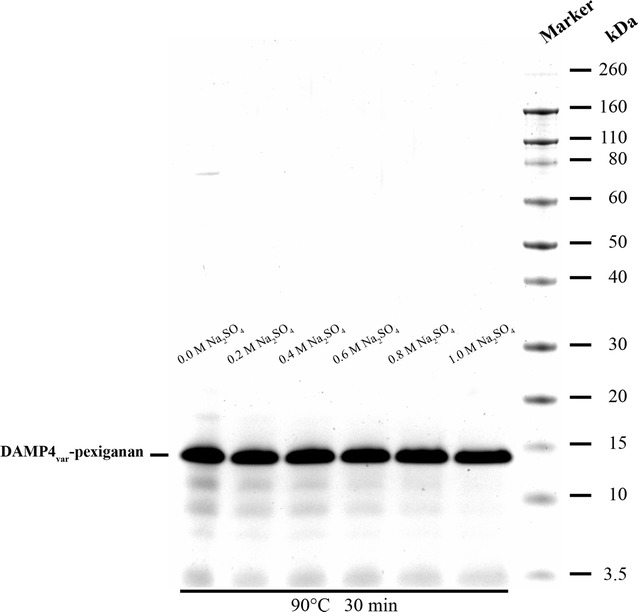
Effect of Na_2_SO_4_ concentrations (0–1 M) on the precipitation of protein contaminants at 90 °C as shown by SDS–PAGE gel

Following DNA removal, the protein contaminants were precipitated by the salting-out effect in the presence of Na_2_SO_4_ (0–1 M) under high temperature (90 °C). Figure 3 shows the SDS-PAGE gel of the supernatants after protein-contaminant precipitation at 90 °C in the presence of Na_2_SO_4_ at different concentrations. In the absence of Na_2_SO_4_, heating the protein mixture at 90 °C for 30 min was unable to remove the protein contaminants completely. Except for the band that appeared at 3.5 kDa in all lanes, which represents residual PEI in the bulk phase after the DNA removal step, only the DAMP4_var_-pexiganan protein band appeared on the SDS-PAGE gel at 1 M Na_2_SO_4_ giving higher protein purity than the other Na_2_SO_4_ concentrations (< 1 M) (Fig. 3).

### Isolation of DAMP4_var_-pexiganan protein

**Fig. 4:**
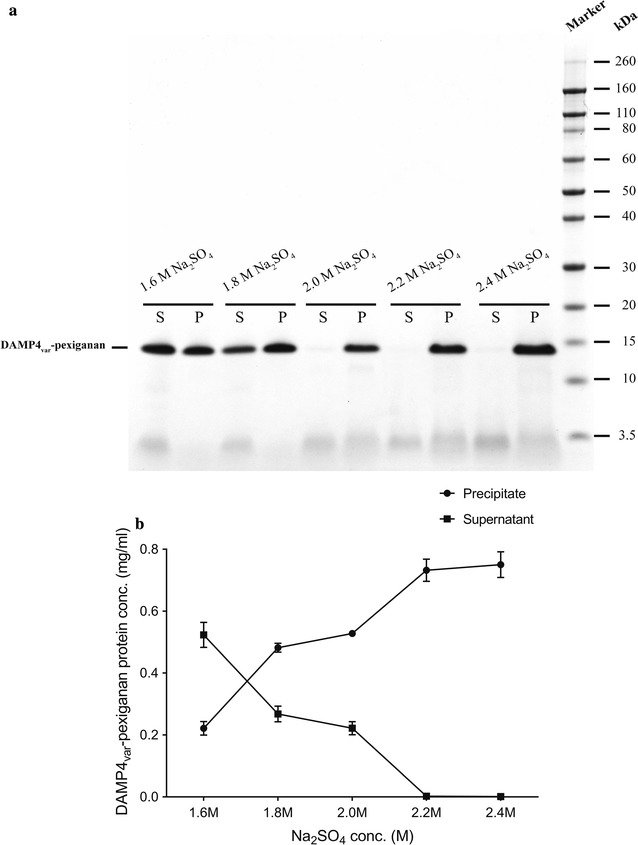
Effect of Na_2_SO_4_ concentrations (1.6–2.4 M) on the solubility of DAMP4_var_-pexiganan protein. **a** SDS–PAGE analysis (S, supernatant; P, precipitate). **b** The concentrations of DAMP4_var_-pexiganan protein in both the supernatant and the solubilized precipitate

Subsequent to the precipitation of protein contaminants, the ionic strength in bulk solution was further increased by adding more Na_2_SO_4_ (1.6–2.4 M), in which case the DAMP4_var_-pexiganan proteins start to precipitate. Next, the precipitates of DAMP4_var_-pexiganan protein were collected via centrifugation and re-solubilized in the solubilizing buffer. Figure 4 shows the effect of Na_2_SO_4_ concentrations on the precipitation of DAMP4_var_-pexiganan proteins. As shown in the SDS-PAGE gel (Fig. 4a), it is clear that increasing the concentrations of Na_2_SO_4_ (1.6–2.4 M) reduced the concentrations of DAMP4_var_-pexiganan protein in the supernatants. We also measured the concentrations of DAMP4_var_-pexiganan protein in the supernatants and in the re-solubilized precipitates using RP-HPLC (Fig. 4b). As the concentrations of Na_2_SO_4_ were increased from 1.6 to 2.4 M, the concentrations of the re-solubilized DAMP4_var_-pexiganan protein increased from 0.22 to 0.74 mg/mL (Fig. 4b). Additionally, no DAMP4_var_-pexiganan protein was detected in the supernatant with 2.2 and 2.4 M Na_2_SO_4_, indicating that 2.2 M Na_2_SO_4_ was sufficient to precipitate all DAMP4_var_-pexiganan protein from the bulk phase.

**Fig. 5:**
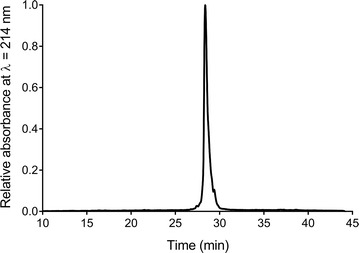
Characterization of DAMP4_var_-pexiganan protein after the purification process as determined by using RP-HPLC

Table 1 shows the mass balance of the whole process of the purification of DAMP4_var_-pexiganan proteins. This purification method can achieve a final yield of 24.0 mg DAMP4_var_-pexiganan proteins from 800 mL cell culture with a final cultivation OD_600_ ~ 2, which accounts for 72.9% of protein recovery. The RP-HPLC result showed that the resultant DAMP4_var_-pexiganan protein was very pure as indicated by the single peak at the retention time of 28.3 min (Fig. 5).

**Table 1:** Mass balance of the downstream processing of DAMP4_var_-pexiganan protein

Process	Protein yield (mg)^a^	Protein recovery (%)
Cell lysis	32.9 ± 0.3	100
DNA removal	30.2 ± 0.2	91.8 ± 0.2
Protein-contaminant precipitation	28.2 ± 0.9	85.8 ± 1.9
DAMP4_var_-pexiganan isolation^b^	24.7 ± 0.5	75.0 ± 1.0
Buffer exchange	24.0 ± 0.6	72.9 ± 1.0

^a^The protein yield was measured by the area of the peak shown in RP-HPLC, except that the protein after cell lysis step was calculated based on SDS-PAGE gel using ImageJ software (Wibowo et al. 2017)

^b^The yield of DAMP4_var_-pexiganan protein after isolation was measured after rinsing the protein precipitate against rinsing buffer (25 mM Tris–HCl, 1 M NaCl, pH 8, 2.2 M Na_2_SO_4_) and then solubilized in solubilizing buffer (25 mM Tris–HCl, 1 M NaCl, pH 8)

### Isolation of pexiganan peptide

**Fig. 6:**
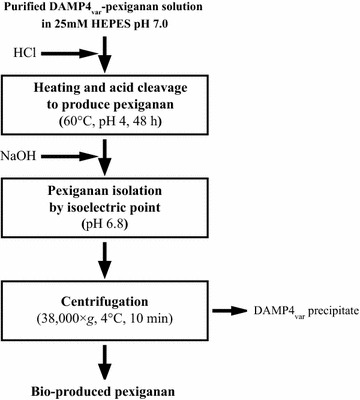
Process flow diagram of the cleavage of pexiganan peptide from the protein carrier DAMP4_var_

**Fig. 7:**
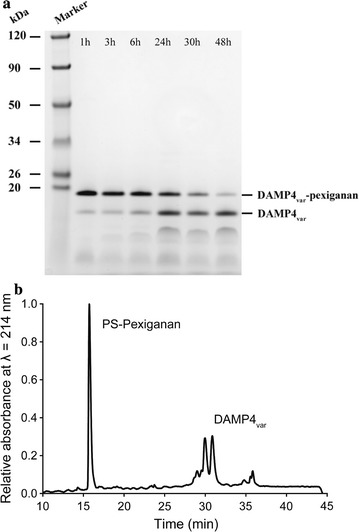
**a** Effect of incubation time on the cleavage of DAMP4_var_-pexiganan protein to yield the bio-produced pexiganan peptide. **b** RP-HPLC characterization of the solution composition after the cleavage and subsequent isoelectric precipitation of DAMP4_var_ protein

A preliminary test demonstrated that the full-length DAMP4_var_-pexiganan protein did not have antimicrobial activity (data not shown). Therefore, the protein was cleaved to yield bio-produced pexiganan peptide (GIGKFLKKAKKFGKAFVKILKKHH) (Fig. 6). By incubating the protein solution at pH 4 and 60 °C, the cleavage occurred between D and P residues in the DPS linker of DAMP4_var_-pexiganan protein that links the DAMP4_var_ protein carrier and the pexiganan peptide (Dimitrijev-Dwyer et al. 2012). The cleavage of DAMP4_var_-pexiganan protein produced DAMP4_var_ (theoretical *MW* 11.2 kDa) and PS-pexiganan (theoretical *MW* 2.9 kDa) at a 1:1 ratio. Figure 7a shows the SDS-PAGE gel result of the cleavage process at different incubation periods. Although the SDS-PAGE gel cannot present the band of pexiganan peptide due to its low molecular weight, it is clear that the DAMP4_var_ protein band appeared on the gel after 1 h incubation and became darker as the incubation time increased. Meanwhile, the DAMP4_var_-pexiganan protein band became lighter with prolonged incubation time (Fig. 7a). These results confirm the successful cleavage at the D-P bond, and most DAMP4_var_-pexiganan proteins could be cleaved over a period of 48 h as confirmed by RP-HPLC (Fig. 7b).

The bio-produced pexiganan peptide was separated from the DAMP4_var_ protein by precipitation based on the differences in their isoelectric points. The theoretical isoelectric points (p*I*) of DAMP4_var_ protein and PS-pexiganan peptide are 6.8 and 11.2, respectively. By adjusting the solution pH to 6.8, the DAMP4_var_ protein precipitated and the PS-pexiganan peptide remained in the bulk phase. The resultant supernatants after centrifugation were analyzed by RP-HPLC which showed two peaks at retention time of 15 and 30 min (Fig. 7). Based on the previous characterization study on the cleavage of DAMP4_var_-pexiganan protein (Zhao et al. 2015), we confirmed that the peaks at 15 and 30 min correspond to PS-pexiganan peptide and DAMP4_var_ protein, respectively, indicating the successful cleavage of PS-pexiganan peptide. A yield of 3.2 mg PS-pexiganan peptides was achieved from 800 mL cell culture (OD_600_ ~ 2) with a 62% of recovery. After the desalting process, the final yield of PS-pexiganan was 1.6 mg, achieving 31% of overall recovery.

### Antimicrobial activity of pexiganan

**Fig. 8:**
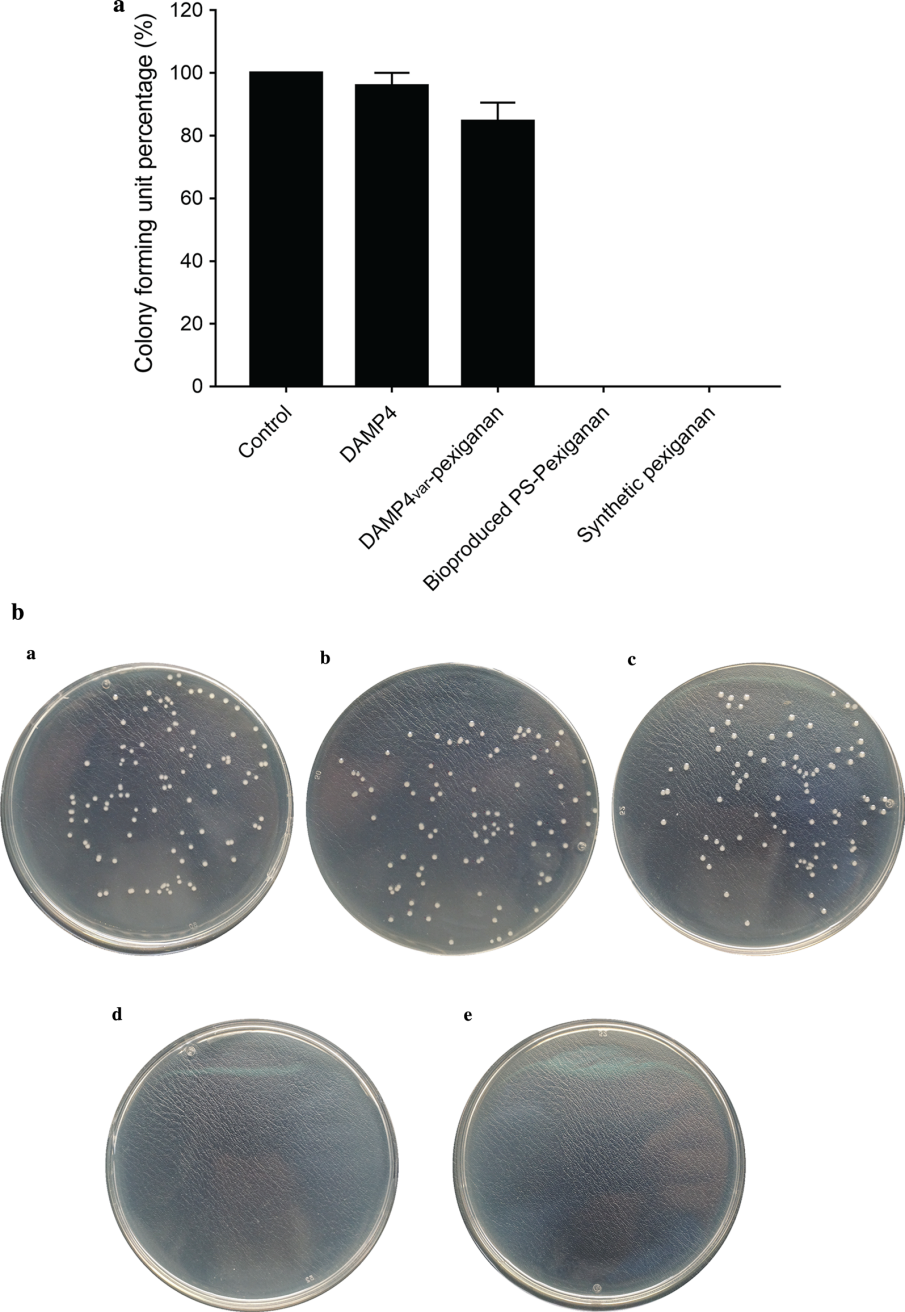
Antimicrobial activity of the bio-produced pexiganan peptide against *E. coli* ATCC^®^ 25922™ in comparison with controls as determined by the minimum bactericidal concentration (MBC) method. **a** The percentages of viable cells after MBC tests. **b** Photographs of *E. coli* growth on agar plates containing of: (a) water; (b) DAMP4 protein; (c) DAMP4_var_-pexiganan protein; (d) synthetic pexiganan peptide; and (e) bio-produced pexiganan peptide. The concentration of all protein/peptide samples was 16 µg/mL

The antimicrobial activity of the bio-produced PS-pexiganan peptide was confirmed by testing against *E. coli* ATCC^®^ 25922™ using minimum bactericidal concentration (MBC) in comparison with the synthetic pexiganan peptide, DAMP4 protein, and DAMP4_var_-pexiganan protein as controls (final concentrations of 1 to 32 µg/mL for each protein/peptide sample). *E. coli* ATCC^®^ 25922™ showed bactericidal response to both the synthetic pexiganan and the bio-produced PS-pexiganan as higher concentrations resulted in less colonies on the plates (Additional file 1: Figs. S1–S5). As shown in Fig. 8, no bacterial growth occurred on the plates after treatment when the concentration of synthetic pexiganan was greater than or equal to 16 µg/mL, indicating the MBC value of 16 µg/mL for the synthetic pexiganan. This result is consistent with previous antimicrobial studies on pexiganan peptide (Ge et al. 1999). Also, no bacterial colonies were observed on the plates after treatment with the bio-produced PS-pexiganan of 16 µg/mL and above. This result confirms that the bio-produced PS-pexiganan possesses the same antimicrobial properties as the synthetic pexiganan. In contrast, the DAMP4 and DAMP4_var_-pexiganan proteins did not exhibit antimicrobial activities, since the control studies on DAMP4 and DAMP4_var_-pexiganan showed the colonies of *E. coli* similar to the control group without any treatment.

## Discussion

**Fig. 9:**
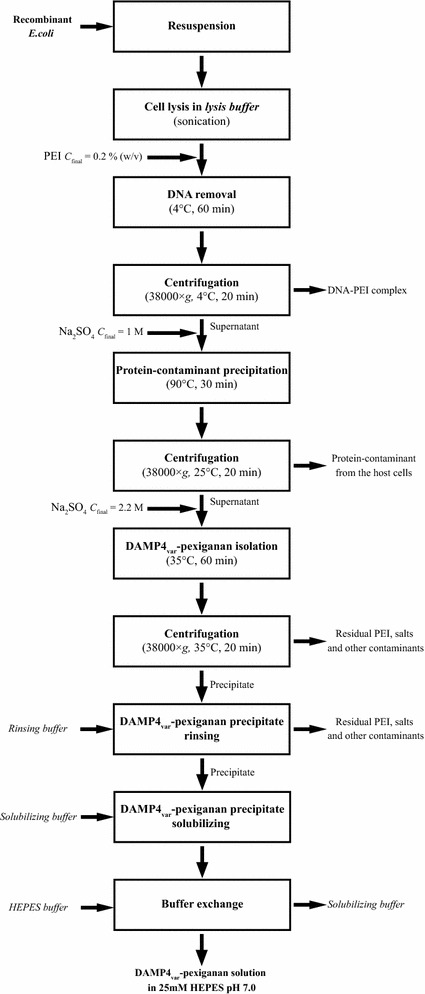
Process flow diagram of the purification of DAMP4_var_-pexiganan protein

This paper presents a new downstream processing method to produce antimicrobial peptides from recombinant *E. coli* based on selective thermochemical lysis (Dwyer et al. 2014) and precipitation (Wibowo et al. 2017) methods. This method relies on the superior thermal and chemical stability of a four helix bundle protein DAMP4. A fusion protein DAMP4_var_-pexiganan was formed by fusing a variant of DAMP4 protein and an antimicrobial peptide pexiganan. This fusion protein could be purified via a selective thermochemical precipitation using the optimized parameters (Fig. 9).

Removal of DNA contaminants is important in downstream processes as their presence causes increased protein solution viscosity and reduced protein functionalities due to their association with proteins via electrostatic interactions (Wyman et al. 1997). Addition of poly(ethyleneimine) (PEI) has been demonstrated to be effective to remove DNA through the formation of charge-neutralization complexes depending on ionic strength of the bulk solution (Burgess 1991; DeWalt et al. 2003). In the step of DNA removal, efficiency of DNA precipitation are influenced by NaCl and PEI concentrations. PEI concentration of 0.2% in the presence of 1 M NaCl facilitated 99% of DNA removal from crude cell lysates. At NaCl concentrations of less than 1 M, the proteins including DAMP4_var_-pexiganan protein electrostatically attached to DNA could co-precipitate along with the PEI–DNA complexes, but further increase of the NaCl concentration inhibited the interaction between DNA and protein (Fig. 1). This is likely due to the association between Na^+^ in the solution and PO_4_^3−^ in the DNA backbone at a minimum NaCl concentration of 1.04 M (Antila et al. 2015), leading to screening of the negatively charged DNA. Thus, high concentrations of NaCl (> 1 M) greatly reduced the electrostatic interaction of DNA and PEI, prevented the formation of PEI–DNA complexes and hence increased the concentration of DNA in the bulk aqueous phase.

The results of the optimization of PEI concentrations (Fig. 2) indicated that increasing PEI concentrations (0.05–0.2%) effectively reduced DNA concentrations in bulk phase due to the formation of PEI–DNA complexes in a form of precipitates. However, further increased of PEI concentrations (> 0.2%) caused the formation of the positively-charged PEI–DNA complexes, which hindered the aggregation due to electrostatic repulsion between the complexes (Ogris et al. 1998).

In the next step, most protein-contaminants were precipitated at 90 °C in the presence of 1 M Na_2_SO_4_ (Fig. 3), while DAMP4_var_-pexiganan protein remained stable and soluble. Na_2_SO_4_ is a kosmotropic salt that is able to withdraw water molecules from proteins, making their hydrophobic interactions more dominance and thus resulting in the protein precipitation. In addition, high temperature unfolds proteins leading to the protein aggregation by the exposure of their hydrophobic residues. The synergistic effect of the high concentration of Na_2_SO_4_ and high temperature precipitated most protein contaminants. However, DAMP4_var_-pexiganan proteins were still soluble and stable under these conditions. This is due to the four helical bundle structure of DAMP4 that interlocks the hydrophobic residues at the protein core, thus giving rise to the unique stability under high temperature and high salt concentration (Dwyer et al. 2014).

By further increasing the concentration of Na_2_SO_4_ up to 2.2 M, DAMP4_var_-pexiganan could be reversibly isolated because of salting-out effect (Fig. 4). The addition of solubilizing buffer (Fig. 9) simply re-solubilized the DAMP4_var_-pexiganan protein precipitates with a purity higher than the purity of DAMP4_var_-pexiganan purified using previous methods (Zhao et al. 2015) as suggested by the RP-HPLC result (Fig. 5). The yield of DAMP4_var_-pexiganan protein purified in this study is increased 200% of the yield from the previous purification method based on thermal treatment (Dwyer et al. 2014).

Unlike most peptide cleavage that uses, for example, hazardous cyanogen bromide (Zhou et al. 2005) or expensive enzyme (Huang et al. 2006), the DPS linker within DAMP4_var_-pexiganan protein provides an easily achievable cleavage method to produce the pexiganan via acid-cleavage between D and P residues in the DPS linker of the fusion protein under a mild heating condition (60 °C, pH 4, 48 h) (Dimitrijev-Dwyer et al. 2012) (Fig. 6). The bio-produced PS-pexiganan peptide (p*I* 11.2) can then be simply separated from DAMP4_var_ protein (p*I* 6.8) based on the differences in their isoelectric points (Fig. 7). By adjusting the solution pH to 6.8, the number of positive and negative charges on DAMP4_var_ protein is nearly equal and thus prone to undergo precipitation. Meanwhile, the bio-produced peptides were still soluble due to their ionic repulsion at pH 6.8.

The pexiganan peptide recombinantly produced in this work (MBC ≈ 16 µg/mL) showed an effective antimicrobial activity similar to the chemically-synthesized pexiganan peptide (MBC ≈ 16 µg/mL) (Fig. 8). Previous work on the antimicrobial mechanism of pexiganan has revealed that the antimicrobial activity of pexiganan results from the unrepairable damage of bacteria membrane. Pexiganan peptides associate with the lipid head group of membranes and induce toroidal-pores in the membrane at a threshold concentration (Yang et al. 2001). Furthermore, the ability of the bio-produced pexiganan peptide in successfully killing *E. coli* cells confirmed that there is no adverse effects due to the bioprocess simplification.

This new downstream processing method is superior to traditional thermal purification methods in terms of yield and purity (Dwyer et al. 2014; Zhao et al. 2015), and more competitive than chromatographic methods in terms of production cost as compared to the previous purification processes of producing antimicrobial pexiganan peptide (Jang et al. 2009). We expect that this new method could offer a cost-effective and high-yield approach which can be easily generalized and adapted to recover and purify other antimicrobial peptides from microbial cell factories. It should be noted that the described methods might not be suitable for facilitating the purification of large proteins such as enzymes, as these purification conditions might change the tertiary conformational structures of the proteins which can result in the loss of protein functionalities.

## Additional file

**Additional file 1: Figure S1.** Photographs of *E. coli* growth on agar plates containing of: (a) water; (b) DAMP4 protein; (c) DAMP4var-pexiganan protein; (d) synthetic pexiganan peptide; and (e) bio-produced pexiganan peptide. The concentration of all protein/peptide samples was 1 μg/mL. **Figure S2.** Photographs of *E. coli* growth on agar plates containing of: (a) water; (b) DAMP4 protein; (c) DAMP4var-pexiganan protein; (d) synthetic pexiganan peptide; and (e) bio-produced pexiganan peptide. The concentration of all protein/peptide samples was 2 μg/mL. **Figure S3.** Photographs of *E. coli* growth on agar plates containing of: (a) water; (b) DAMP4 protein; (c) DAMP4var-pexiganan protein; (d) synthetic pexiganan peptide; and (e) bio-produced pexiganan peptide. The concentration of all protein/peptide samples was 4 μg/mL. **Figure S4.** Photographs of *E. coli* growth on agar plates containing of: (a) water; (b) DAMP4 protein; (c) DAMP4var-pexiganan protein; (d) synthetic pexiganan peptide; and (e) bio-produced pexiganan peptide. The concentration of all protein/peptide samples was 8 μg/mL. **Figure S5.** Photographs of *E. coli* growth on agar plates containing of: (a) water; (b) DAMP4 protein; (c) DAMP4var-pexiganan protein; (d) synthetic pexiganan peptide; and (e) bio-produced pexiganan peptide. The concentration of all protein/peptide samples was 32 μg/mL.

## Abbreviations

2YT: 2× yeast extract and tryptone
AMPs: antimicrobial peptides
ATCC: American type culture collection
CFU: colony-forming unit
HEPES: 4-(2-hydroxyethyl)-1-piperazineethanesulfonic acid
LB: Luria-Bertani
MBC: minimum bactericidal concentration
MH: Mueller–Hinton
*MW*: molecular weight
OD_600_: optical density at a wavelength of 600 nm
PEI: poly(ethyleneimine)
p*I*: isoelectric point
RP-HPLC: reversed-phase high-performance liquid chromatography
SDS-PAGE: sodium dodecyl sulfate polyacrylamide gel electrophoresis
TFA: trifluoroacetic acid
