# Supplementary material for: Cost-effective downstream processing of recombinantly produced pexiganan peptide and its antimicrobial activity
Source: AMB Express. 2018 Jan 24;8:6. doi: 10.1186/s13568-018-0541-3 (PMC5783979; doi:10.1186/s13568-018-0541-3)
Supplement: Supplementary file 1 — Additional file 1: Figure S1. Photographs of E. coli growth on agar plates containing of: (a) water; (b) DAMP4 protein; (c) DAMP4var-pexiganan protein; (d) synthetic pexiganan peptide; and (e) bio-produced pexiganan peptide. The concentration of all protein/peptide samples was 1 μg/mL. Figure S2. Photographs of E. coli growth on agar plates containing of: (a) water; (b) DAMP4 protein; (c) DAMP4var-pexiganan protein; (d) synthetic pexiganan peptide; and (e) bio-produced pexiganan peptide. The concentration of all protein/peptide samples was 2 μg/mL. Figure S3. Photographs of E. coli growth on agar plates containing of: (a) water; (b) DAMP4 protein; (c) DAMP4var-pexiganan protein; (d) synthetic pexiganan peptide; and (e) bio-produced pexiganan peptide. The concentration of all protein/peptide samples was 4 μg/mL. Figure S4. Photographs of E. coli growth on agar plates containing of: (a) water; (b) DAMP4 protein; (c) DAMP4var-pexiganan protein; (d) synthetic pexiganan peptide; and (e) bio-produced pexiganan peptide. The concentration of all protein/peptide samples was 8 μg/mL. Figure S5. Photographs of E. coli growth on agar plates containing of: (a) water; (b) DAMP4 protein; (c) DAMP4var-pexiganan protein; (d) synthetic pexiganan peptide; and (e) bio-produced pexiganan peptide. The concentration of all protein/peptide samples was 32 μg/mL. [file 13568_2018_541_MOESM1_ESM.pdf]

# Supporting Information

## Cost-effective downstream processing of recombinantly produced pexiganan peptide and its antimicrobial activity

Baode Sun[1], David Wibowo[1*], Anton P.J. Middelberg[2], Chun-Xia Zhao[1*]

[1]*Australian Institute for Bioengineering and Nanotechnology, The University of Queensland, St Lucia, QLD 4072, Australia.* [2]*Faculty of Engineering, Computer, and Mathematical Sciences, The University of Adelaide, Adelaide, SA 5005, Australia.*

*Correspondence to: D. Wibowo; C.-X Zhao

Telephones: +61-7-3346-4267 (D. Wibowo); +61-7-3346-4263 (C.-X Zhao)

E-mails: d.wibowo@uq.edu.au (D. Wibowo); z.chunxia@uq.edu.au (C.-X Zhao)

ORCIDs: 0000-0001-6919-3355 (D. Wibowo); 0000-0002-3365-3759 (C.-X Zhao)

**Acknowledgments**

This research was supported by the Australian Research Council (ARC) under Discovery Project (DP150100798). C.-X.Z. acknowledges financial support from the award of the ARC Future Fellowship (FT140100726).

## Figures Legends

**Figure S1.** Photographs of *E. coli* growth on agar plates containing of: (a) water; (b) DAMP4 protein; (c) $DAMP4_{var}$-pexiganan protein; (d) synthetic pexiganan peptide; and (e) bio-produced pexiganan peptide. The concentration of all protein/peptide samples was 1 μg/mL.

**Figure S2.** Photographs of *E. coli* growth on agar plates containing of: (a) water; (b) DAMP4 protein; (c) $DAMP4_{var}$-pexiganan protein; (d) synthetic pexiganan peptide; and (e) bio-produced pexiganan peptide. The concentration of all protein/peptide samples was 2 μg/mL.

**Figure S3.** Photographs of *E. coli* growth on agar plates containing of: (a) water; (b) DAMP4 protein; (c) $DAMP4_{var}$-pexiganan protein; (d) synthetic pexiganan peptide; and (e) bio-produced pexiganan peptide. The concentration of all protein/peptide samples was 4 μg/mL.

**Figure S4.** Photographs of *E. coli* growth on agar plates containing of: (a) water; (b) DAMP4 protein; (c) $DAMP4_{var}$-pexiganan protein; (d) synthetic pexiganan peptide; and (e) bio-produced pexiganan peptide. The concentration of all protein/peptide samples was 8 μg/mL.

**Figure S5.** Photographs of *E. coli* growth on agar plates containing of: (a) water; (b) DAMP4 protein; (c) $DAMP4_{var}$-pexiganan protein; (d) synthetic pexiganan peptide; and (e) bio-produced pexiganan peptide. The concentration of all protein/peptide samples was 32 μg/mL.

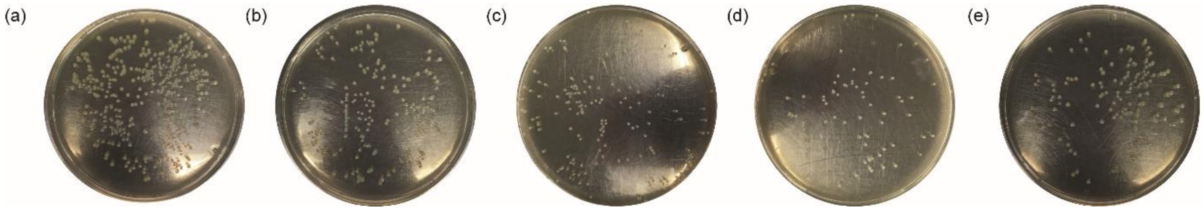


**Figure S1.** Photographs of *E. coli* growth on agar plates containing of: (a) water; (b) DAMP4 protein; (c) $DAMP4_{var}$-pexiganan protein; (d) synthetic pexiganan peptide; and (e) bio-produced pexiganan peptide. The concentration of all protein/peptide samples was 1 μg/mL.

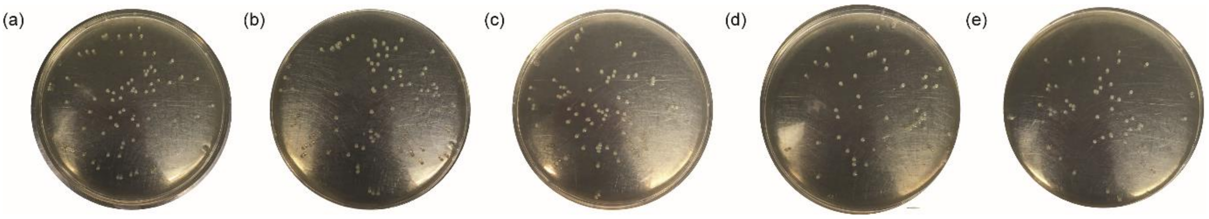


**Figure S2.** Photographs of *E. coli* growth on agar plates containing of: (a) water; (b) DAMP4 protein; (c) $DAMP4_{var}$-pexiganan protein; (d) synthetic pexiganan peptide; and (e) bio-produced pexiganan peptide. The concentration of all protein/peptide samples was 2 μg/mL.

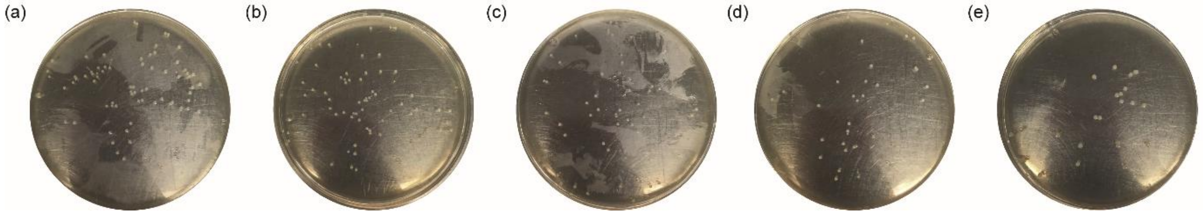

**Figure S3.** Photographs of *E. coli* growth on agar plates containing of: (a) water; (b) DAMP4 protein; (c) $DAMP4_{var}$-pexiganan protein; (d) synthetic pexiganan peptide; and (e) bio-produced pexiganan peptide. The concentration of all protein/peptide samples was 4 µg/mL.

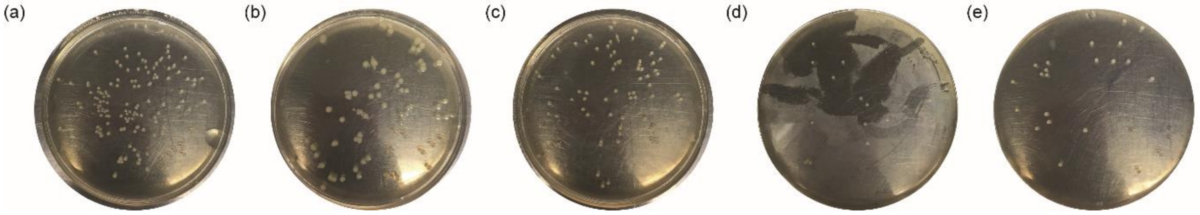


**Figure S4.** Photographs of *E. coli* growth on agar plates containing of: (a) water; (b) DAMP4 protein; (c) $DAMP4_{var}$-pexiganan protein; (d) synthetic pexiganan peptide; and (e) bio-produced pexiganan peptide. The concentration of all protein/peptide samples was 8 μg/mL.

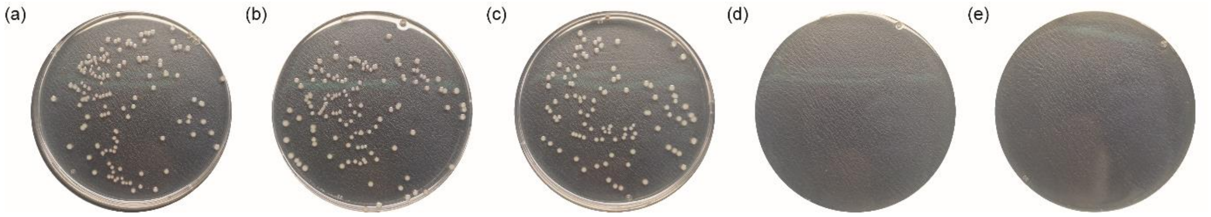


**Figure S5.** Photographs of *E. coli* growth on agar plates containing of: (a) water; (b) DAMP4 protein; (c) DAMP4$_{var}$-pexiganan protein; (d) synthetic pexiganan peptide; and (e) bio-produced pexiganan peptide. The concentration of all protein/peptide samples was 32 μg/mL.
